# Supplementary material for: From sequence to enzyme mechanism using multi-label machine learning
Source: BMC Bioinformatics. 2014 May 19;15:150. doi: 10.1186/1471-2105-15-150 (PMC4229970; doi:10.1186/1471-2105-15-150)
Supplement: Additional file 2 — Java code of ml2db. Additional file ml2db_code.tar.gz contains the Java source code to run the multi-label machine learning experiments and save the results to database. The code’s Javadoc is included. [file 1471-2105-15-150-S2.zip › additional file 2/ml2db/ml2db/doc/mulan/classifier/meta/RAkELd.html]

RAkELd


JavaScript is disabled on your browser.


- Overview
- Package
- Class
- Use
- Tree
- Deprecated
- Index
- Help

- Prev Class
- Next Class

- Frames
- No Frames

- All Classes

- Summary:
- Nested |
- Field |
- Constr |
- Method

- Detail:
- Field |
- Constr |
- Method


mulan.classifier.meta

## Class RAkELd

- java.lang.Object
- - mulan.classifier.MultiLabelLearnerBase
  - - mulan.classifier.meta.MultiLabelMetaLearner
    - - mulan.classifier.meta.RAkELd

- All Implemented Interfaces:
  :   java.io.Serializable, MultiLabelLearner, weka.core.TechnicalInformationHandler

  ---

    

  ```
  public class RAkELd
  extends MultiLabelMetaLearner
  ```

  Class implementing a generalized version of the RAkEL-d (RAndom k-labELsets) algorithm with disjoint labelsets. For more information, see

  Version:
  :   2012.07.16

  Author:
  :   Ioannis Katakis, Grigorios Tsoumakas

  See Also:
  :   Serialized Form

- - ### Constructor Summary

    Constructors

    Constructor and Description

    `RAkELd()`

    Default constructor

    `RAkELd(MultiLabelLearner baseLearner)`

    Construct a new instance based on the given multi-label learner

    `RAkELd(MultiLabelLearner baseLearner,
    int subset)`

    Constructs a new instance based on the given multi-label learner and
    size of subset
  - ### Method Summary

    Methods

    | Modifier and Type | Method and Description |
    | --- | --- |
    | `int` | `getSizeOfSubset()` Returns the size of the subsets |
    | `weka.core.TechnicalInformation` | `getTechnicalInformation()` Returns an instance of a TechnicalInformation object, containing detailed information about the technical background of this class, e.g., paper reference or book this class is based on. |
    | `java.lang.String` | `globalInfo()` Returns a string describing classifier |
    | `void` | `setSeed(int x)` Sets the seed for random number generation |
    | `void` | `setSizeOfSubset(int size)` Sets the size of the subsets |
    | `void` | `updateClassifier(MultiLabelInstances mlTrainData, int model)` Updates the current ensemble by training a specific classifier |

    - ### Methods inherited from class mulan.classifier.meta.MultiLabelMetaLearner

      `getBaseLearner`
    - ### Methods inherited from class mulan.classifier.MultiLabelLearnerBase

      `build, getDebug, isUpdatable, makeCopy, makePrediction, setDebug`
    - ### Methods inherited from class java.lang.Object

      `equals, getClass, hashCode, notify, notifyAll, toString, wait, wait, wait`

- - ### Constructor Detail


    - #### RAkELd

      ```
      public RAkELd()
      ```

      Default constructor


    - #### RAkELd

      ```
      public RAkELd(MultiLabelLearner baseLearner)
      ```

      Construct a new instance based on the given multi-label learner

      Parameters:
      :   `baseLearner` - a multi-label learner


    - #### RAkELd

      ```
      public RAkELd(MultiLabelLearner baseLearner,
            int subset)
      ```

      Constructs a new instance based on the given multi-label learner and
      size of subset

      Parameters:
      :   `baseLearner` - the multi-label learner
      :   `subset` - the size of the subset
  - ### Method Detail


    - #### getTechnicalInformation

      ```
      public weka.core.TechnicalInformation getTechnicalInformation()
      ```

      Returns an instance of a TechnicalInformation object, containing
      detailed information about the technical background of this class,
      e.g., paper reference or book this class is based on.

      **Specified by:**
      :   `getTechnicalInformation` in interface `weka.core.TechnicalInformationHandler`

      **Specified by:**
      :   `getTechnicalInformation` in class `MultiLabelLearnerBase`

      Returns:
      :   the technical information about this class


    - #### setSeed

      ```
      public void setSeed(int x)
      ```

      Sets the seed for random number generation

      Parameters:
      :   `x` - the seed


    - #### setSizeOfSubset

      ```
      public void setSizeOfSubset(int size)
      ```

      Sets the size of the subsets

      Parameters:
      :   `size` - size of subsets


    - #### getSizeOfSubset

      ```
      public int getSizeOfSubset()
      ```

      Returns the size of the subsets

      Returns:
      :   the size of the subsets


    - #### updateClassifier

      ```
      public void updateClassifier(MultiLabelInstances mlTrainData,
                          int model)
                            throws java.lang.Exception
      ```

      Updates the current ensemble by training a specific classifier

      Parameters:
      :   `mlTrainData` - the training data
      :   `model` - the model to train

      Throws:
      :   `java.lang.Exception`


    - #### globalInfo

      ```
      public java.lang.String globalInfo()
      ```

      Returns a string describing classifier

      **Specified by:**
      :   `globalInfo` in class `MultiLabelLearnerBase`

      Returns:
      :   a description suitable for displaying


- Overview
- Package
- Class
- Use
- Tree
- Deprecated
- Index
- Help

- Prev Class
- Next Class

- Frames
- No Frames

- All Classes

- Summary:
- Nested |
- Field |
- Constr |
- Method

- Detail:
- Field |
- Constr |
- Method
